# Delayed cervical emphysema after thyroidectomy: a case report and a literature overview

**DOI:** 10.1093/jscr/rjae435

**Published:** 2024-07-12

**Authors:** Kjersti Mevik, Gunnbjørg Tysvær, Torill Solli, Terje Osnes

**Affiliations:** Surgery Department, Nordland Hospital, Parkveien 95, 8092 Bodø, Norway; Surgery Department, Nordland Hospital, Parkveien 95, 8092 Bodø, Norway; Surgery Department, Nordland Hospital, Parkveien 95, 8092 Bodø, Norway; Department of Otorhinolaryngology and Head and Neck Surgery, Oslo University Hospital, Sognsvannsveien 20, 0379 Oslo, Norway; University of Oslo, 0371 Oslo, Norway

**Keywords:** subcutaneous emphysema, controlled negative pressure drain, delayed complications, thyroidectomy

## Abstract

Thyroidectomy is a surgery performed due to both benign and malign diseases in the thyroid. The overall complication rate is low, where most of them will appear within the first 24 hours after surgery. However, severe complications can occur as late as 14 days postsurgery. A woman in her late 30’s underwent total thyroidectomy due to Graves' disease. There were no complications until she presented with swelling on her neck 10 days after surgery. She was diagnosed with cervical emphysema and treated with a controlled negative pressure drain until there was no more air leakage. We assumed that the emphysema was due to an occult injury of the trachea. Urgent evaluation and hospitalization are needed if the patient presents with swelling in the neck after thyroidectomy. Surgeons should be aware of this delayed complication, so they are able to inform and manage their patients accordingly.

## Introduction

Total thyroidectomy is considered a low-risk procedure with mostly minor complications which will usually appear within the first 24 hours. Delayed complications can occur up to, but not limited to, 2 weeks after the surgery but are very rare. Such complications can be lymphatic fluid, leakage (chyle), fistulas, infections, and emphysema [1, 2]. Emphysema due to iatrogenic injury after thyroidectomy has been reported as low as 0.06%, and most of these injuries are occult [1]. Such injury could present with developing emphysema causing trouble of breathing for the patient. We here present a case of cervical emphysema after thyroidectomy.

## Case report

The patient had relapse of Graves' disease and therefore definitive treatment with total thyroidectomy was decided upon. Her symptoms included tremors, fatigue, and palpitations without eye symptoms. Preoperative the patient was euthyroid. Her regular medications included varenicline and carbimazole. She smoked, approximately 2 cigarettes per day. Previously, the patient had undergone two caesarean sections and was diagnosed with gestational diabetes during her last pregnancy 6 years earlier. Otherwise, she was healthy and fully employed.

**Figure 1:**
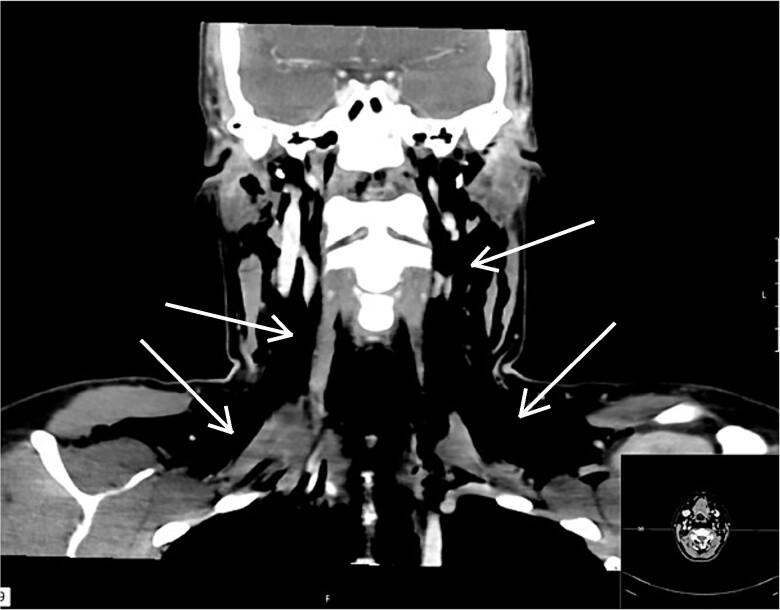
Frontal CT images of the collum. Arrows showing air pockets.

**Figure 2:**
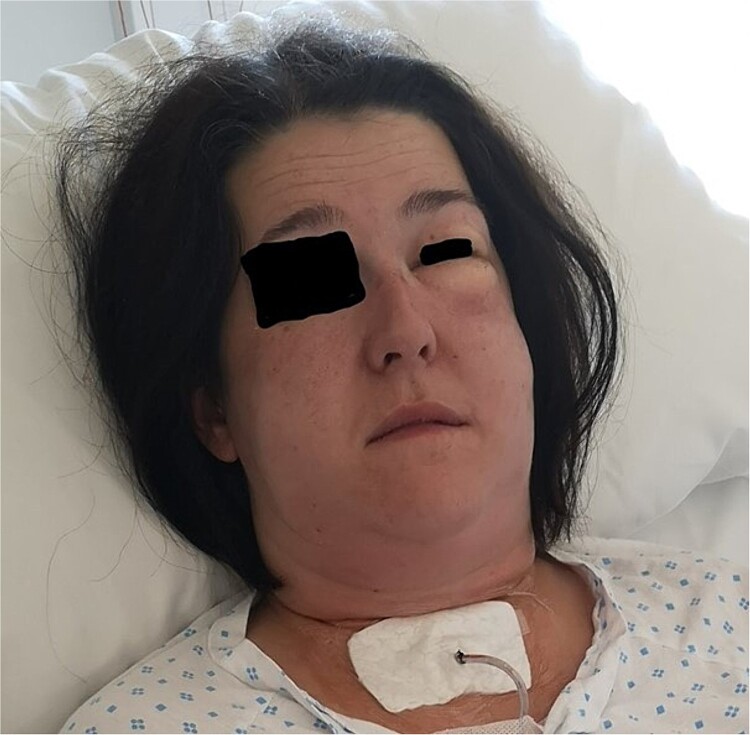
Patient with drain placed in the wound cavity and excessive emphysema on the left side. Photo: Lars Helge Kristensen.

The surgery was performed without any difficulties and diathermy was used for electrocoagulation, and Ligasure was used for tissue sealing. Hemostatic material, Cellistypt®, was placed on both sides of the trachea after the thyroid was removed. No drain was inserted. She was discharged home in good condition without any complications. Carbimazole was discontinued, and the patient started on 100 mcg of levothyroxine daily. The patient was on a routine 2-week sick leave from work. The postoperative course was uneventful until the night of Day 10, when the patient experienced nocturnal coughing bouts followed by throat pain, voice changes, and a sensation of throat fullness. Upon examination at the doctor's office the following day, swelling was observed laterally on the neck bilaterally, most pronounced on the left side from upper thorax to the temple. The patient reported that the swelling had increased since the past hours. She reported increasing pain in the jaw/neck area and noted bubbling and crackling sensations in the throat. She was taken immediately to the hospital due to a threatened airway. The surgical incision appeared unremarkable without any signs of infections. A CT scan was performed revealing extensive subcutaneous emphysema, including pneumomediastinum and pneumopericardium without any defect in the trachea or oesophagus (Fig. 1). We consulted with the national hospital and were advised to only insert a drain and not to search for possible damage to the trachea, as this could cause more harm and the hole may have already begun to close. When the incision was opened under general anesthesia, air immediately escaped, and a drain was placed into the wound cavity without any search for a defect. The skin was closed tightly around the drain to prevent air leakage and displacement. The drain was connected to active suction at −8 mm Hg which was gradually reduced until −1 mm Hg on Day 9 and removed on Day 11 (Fig. 2). She was administered broad-spectrum antibiotics according to Norwegian guidelines and awoken the day after drain placement and discharged on Day 11. At the follow-up 16 days later, she described some discomfort in her throat, feeling dry, and sore but elsewhere she was in good health, and all emphysema had been absorbed. She had returned to full-time work.

## Discussion

In cases of recurrent Graves' disease, when antithyroid drugs won’t work, definitive treatment is recommended either through radioiodine therapy or surgery. Both options are considered equivalent as long as there are no contraindications. The surgery is performed as standard operative technique including meticulous dissection to identify the recurrent laryngeal nerve and parathyroid glands to avoid damage to these structures [3]. There is no evidence that the placement of a drain postoperatively reduces the risk of complications. On the contrary, a drain may increase postoperative pain and the need for analgesics [4]. The most common transient postoperative complications following thyroidectomy are hypocalcaemia due to reduced or damaged circulation to the parathyroid glands (incidence 7–49%) [5], or damage to the recurrent laryngeal nerve resulting in vocal cord paralysis (up to 30%), postoperative bleeding (up to 1%), and infection (<1%) [6–8]. Lymphatic fluid (chyle) leakage may also occur, but this is rare without concurrent neck dissection. Bleeding after thyroidectomy typically occurs within the first 24 hours after surgery [9]. Subcutaneous emphysema has also been described in cases of tracheal or laryngeal injury [10]. Hypocalcaemia and nerve damage can also be permanent complications, reported with frequencies of 1–13% and 0.5–5%, respectively [11, 12]. Transient hypocalcaemia due to reduced or damaged circulation to the parathyroid glands (incidence 7–49%) [5], or damage to the recurrent laryngeal nerve resulting in vocal cord paralysis (up to 30%) and postoperative bleeding (up to 1%) [6, 7–9] are the most common complications. Hypocalcaemia and nerve damage can also be permanent complications, reported with frequencies of 1–13% and 0.5–5%, respectively [11, 12]. Most common delayed complications are infections, lymphatic fluid (chyle) leakage, and very rare subcutaneous emphysema due to tracheal or laryngeal injury [10] Swelling of the incision site is common but increasing swelling spreading to other areas or starting at other areas than the incision should be investigated immediately as the patient may develop a threatening airway obstruction. Swelling can be due to bleeding or lymphatic leakage, but it could also be a sign of emphysema. Additionally, infection and abscess formation must always be ruled out. Subcutaneous emphysema is air trapped in the subcutaneous tissue which can occur for different reasons, for example after trauma, surgery, or due to infection. It manifests as sudden swelling, voice changes (nasal voice), sore throat, and in severe cases, dysphagia, pain, and breathing difficulties. Emphysema after neck surgery is very rare but can occur after various procedures in the neck, including tonsillectomy, thyroidectomy, and tracheostomy [13]. If tracheal injury is detected intraoperatively, direct repair is recommended. This may involve suturing, revision with end-to-end anastomosis, or the use of a muscle flap. The method chosen depends on the patient's condition, the location, and the extent of the injury. Additionally, the condition of the tissue is crucial. Small injuries can be closed primarily. For larger defects and/or damaged tissue, revision and muscle interposition are often required. The sternohyoid or sternocleidomastoid muscles are commonly used as flaps over the defect. This tissue adheres well to the trachea and maintains stiffness, resulting in a good functional outcome [1, 14]. In other cases, in addition to excising necrotic tissue, it may also be necessary to excise parts of the trachea with subsequent anastomosis. Such injuries are more commonly seen when there has been resection of a tumor adherent to the trachea or with invasion of the trachea. Occult injuries manifesting as emphysema after the surgery could be managed conservatively with controlled negative pressure drainage suction as used for pneumothorax. Leong *et al*. [15] presented a case where repair with a muscle flap was performed and a drain with low-pressure suction was used. It is recommended to keep the active drain until there has been no leakage for 24 hours before the drain is closed off and then removed. We assumed that the cause of the subcutaneous emphysema to our patient was a blowout in the trachea during coughing bouts where a portion of the tissue was particularly vulnerable, likely due to the use of electrocoagulation. The electrocoagulation could have caused a small burn injury to the tissue leading to necrosis. When the patient coughed, the pressure raised, causing a small rupture of the trachea. We concluded that the tracheal defect might not be visible to the naked eye and decided to insert a drain into the wound cavity on the side with the most leakage [15]. The need for active negative suction pressure was also indicated by the presence of pneumomediastinum and pneumopericardium. To prevent the negative pressure from keeping the tracheal opening open, the suction pressure is gradually reduced [12]. Additionally, it is important to inform patients to avoid coughing/irritation that could make it difficult for the hole to close. Increasing swelling spreading to other areas or starting at other areas than the incision should be investigated immediately as the patient may develop a threatening airway obstruction. Subcutaneous emphysema is air trapped in the subcutaneous tissue which can occur for different reasons, e.g. after trauma, surgery, or due to infection. It manifests as sudden swelling, voice changes (nasal voice), sore throat, and in severe cases, dysphagia, pain, and breathing difficulties. Emphysema after neck surgery is very rare but can occur after various procedures in the neck, including tonsillectomy, thyroidectomy, and tracheostomy [13]. If tracheal injury is detected intraoperatively, direct repair is recommended. This may involve suturing, revision with end-to-end anastomosis, or the use of a muscle flap. The method chosen depends on the patient's condition, the location, and the extent of the injury. Additionally, the condition of the tissue is crucial. Small injuries can be closed primarily. For larger defects and/or damaged tissue, revision and muscle interposition are often required. The sternohyoid or sternocleidomastoid muscles are commonly used as flaps over the defect [1, 14]. In other cases, in addition to excising necrotic tissue, it may also be necessary to excise parts of the trachea with subsequent anastomosis. Occult injuries manifesting as emphysema after the surgery could be managed with controlled negative pressure drainage suction as used for pneumothorax. Leong *et al*. [15] presented a case where repair with a muscle flap was performed and a drain with low-pressure suction was used. It is recommended to keep the active drain until there has been no leakage for 24 hours before the drain is closed off and then removed.

We assumed that the cause of the subcutaneous emphysema to our patient was a blowout in the trachea during coughing bouts where a portion of the tissue was particularly vulnerable, likely due to the use of electrocoagulation. The electrocoagulation could have caused a small burn injury to the tissue leading to necrosis. When the patient coughed, the pressure raised, causing a small rupture of the trachea. We decided only to insert a drain into the wound cavity on the side with the most leakage [15]. The need for active negative suction pressure was also indicated by the presence of pneumomediastinum and pneumopericardium. To prevent the negative pressure from keeping the tracheal opening open, the suction pressure is gradually reduced [15]. Additionally, it is important to inform patients to avoid coughing/irritation that could make it difficult for the hole to close. This case demonstrates that this could happen to anyone having a procedure close to the trachea.

## Conclusion

This case shows that surgery performed close to the trachea can cause occult trachea injuries leading to emphysema. This is a rare complication which needs to be treated immediately due to obstructing airway. We urge surgeons who operate close to the trachea to be aware of this complication and its symptoms.

## Data Availability

The data used in this publication is stored in the electronical medical record of the patient.
